# Mycological aspects of onychomycosis in Khuzestan Province, Iran: A shift from dermatophytes towards yeasts

**DOI:** 10.29252/cmm.3.4.26

**Published:** 2017-12

**Authors:** Mahnaz Fatahinia, Sima Jafarpour, Abdollah Rafiei, Simin Taghipour, Koichi Makimura, Ali Rezaei-Matehkolaei

**Affiliations:** 1 Department of Medical Mycology, Faculty of Medicine, Ahvaz Jundishapur University of Medical Sciences, Ahvaz, Iran; 2 Health Research Institute, Infectious and Tropical Diseases Research Center, Ahvaz Jundishapur University of Medical Sciences, Ahvaz, Iran; 3 Department of Medical Parasitology, Faculty of Medicine, Ahvaz Jundishapur University of Medical Sciences, Ahvaz, Iran; 4 Laboratory of Space and Environmental Medicine, Graduate School of Medicine, Teikyo University, Tokyo, Japan; 5 General Medical Education and Research Center, Teikyo University, Tokyo, Japan

**Keywords:** Dermatophyte, Khuzestan, Non-dermatophytic molds, Onychomycosis, Yeast

## Abstract

**Background and Purpose:**

Onychomycosis is fungal infection of the nails with an overall increasing incidence, worldwide. The epidemiological aspects of onychomycosis in Khuzestan, Iran, have not been established. In this study, we aimed to evaluate the clinical and mycological status of fungal nail infection in Khuzestan Province, Iran.

**Materials and Methods:**

The study population included 433 patients (143 males vs. 290 females). Nail samples underwent primary direct microscopy and culture. The isolated yeasts and dermatophytes were then subjected to additional molecular identification by r-DNA ITS-RFLP. Identification of some non-dermatophytic molds (NDMs) and unknown yeasts was accomplished by ITS and beta-tubulin sequencing.

**Results:**

Onychomycosis was confirmed in 154 patients (males: 36.4%; n=56 vs. females: 63.6%; n=98), whose age ranged from 2 to 85 years, with the highest prevalence in the age group of 41-50 years. Infection mostly occurred due to yeasts (57.15%), with *Candida albicans* as the most frequent (29.35%) species, followed by *C. parapsilosis* (13.8%) and *C. tropicalis* (4.5%)*.* Dermatophytes were isolated in 38.35% of the cases; the most common isolates were found to be *Trichophyton interdigitale* (21.1%), *Epidermophyton floccosum* (10.5%), *T. rubrum* (5.25%), and *Microsporum canis* (1.5%). NDMs were isolated from 4.5% of the cases with *Aspergillus* spp. as the most common agent. Dermatophytes and NDMs were more frequently seen in toenails, whereas yeasts mostly infected fingernails. Fingernail onychomycosis was significantly more prevalent among females than in males (*P<0.05*).

**Conclusion:**

The study highlights that in Khuzestan province, the causative agents of onychomycosis have shifted from dermatophytes to yeasts.

## Introduction

Onychomycosis is a fungal infection of the fingernails and/or toenails that accounts for approximately 50% of all clinical disorders of the nail and 30% of all superficial mycoses with an overall increasing incidence worldwide. Etiologically, the infection is associated with three groups of keratinophilic fungi, including dermatophytes, non-dermatophytic molds (NDMs), and yeasts. Dermatophytes in *Trichophyton*, *Microsporum*, and *Epidermophyton* genera are the main agents of onychomycosis (tinea unguium), with *T. rubrum* and *T. interdigitale* being the most prevalent species in the world [1, 2]. Less frequently, NDMs in the genera of *Fusarium, Aspergillus*, *Penicillium*, and *Scopulariopsis*, as well as some melanized fungi and yeasts such as *Candida* and *Trichosporon* species can develop this infection [3-7].

Immunosuppression, diabetes, prolonged cortico-steroid therapy, and occlusive footwear are among the factors contributing to onychomycosis [4, 7]. In spite of the unsightly and painful appearance of the affected nails, for a long time, onychomycosis has been regarded as a cosmetic complication of relatively low importance that was hardly noteworthy for elimination. This assumption may have been supported by the negative impacts of high dosage and prolonged treatment related to some initial antifungal medications [3, 4]. 

Today, it is known that this condition is not merely a cosmetic concern because there are many clinical conditions such as psoriasis and lichen planus that mimic onychomycosis [5, 7]. Moreover, onychomycosis is a source of anxiety, inconvenience, embarrassment, as well as detrimental psychosocial and physical effects [3, 4, 8]. The management of this infection consists of antifungal therapy of nail infection, which is often prolonged and accompanied by potential adverse effects; therefore, it is necessary to confirm the clinical diagnosis with laboratory mycological experiments. Likewise, depending on the causative agent, the choice of treatment is variable, indicating the necessity for the exact identification of the fungal etiologic agent [2-4]. This infection has been described in different areas of Iran [9-14], but not in Khuzestan Province. The aim of the present research was to characterize the mycological and epidemiological aspects of onychomycosis in Khuzestan Province, southwest of Iran.

## Materials and Methods

***Patients and Isolates***

In this one-year prospective study performed during September 2015-August 2016, we enrolled 433 clinically suspected cases of onychomycosis (143 males and 290 females) presenting to our medical mycology laboratory (Iran Zamin diagnostic laboratory, Ahvaz, Iran) and outpatient dermatology clinic, affiliated to Faculty of Medicine, Ahvaz Jundishapur University of Medical Sciences, Ahvaz, Iran. 

The demographic data of each patient such as age, gender, and site of infection were recorded using a questionnaire. An ethical code (AJUMS.REC.1393.34) was provided by the Ethics Committee of Ahvaz Jundishapur University of Medical Sciences to conduct the study. The nail surface of each patient was firstly cleaned with 70% alcohol, and the samples were aseptically collected as nail cut, nail clippings, or nail debris. The remaining materials were inoculated onto Sabouraud Dextrose Agar (BD Difco™, Sparks, MD, USA) with chloramphenicol, and with or without cycloheximide. Based on direct microscopic findings, the cultures were incubated at 28ºC or 37°C and checked daily for four weeks (in case of dermatophytes) before being regarded as negative. All isolates regarded as dermatophytes, yeasts, or NDMs in preliminary screening by gross morphology, microscopic or other biochemical properties were subjected to additional molecular identification.

***Identification of the Isolates***

We used PCR-RFLP and sequencing procedures to characterize all dermatophytes, yeasts, and molds at the species level. Firstly, the genomic DNA of each isolate was mechanically extracted by milling of a small amount of fresh colony in the presence of glass beads and the lysis buffer in a homogenizer (SpeedMill PLUS, Analytik Jena, Germany) as previously described [15]. The isolated DNAs were then purified by phenol-chloroform extraction, precipitated with ethanol in the presence of 0.3 M sodium acetate, washed with 70% ethanol, and finally re-suspended in water. The *5.8S* region of *r-DNA* and *its flanking* internal transcribed spacers (ITS1/ITS2) were amplified in dermatophytes and yeasts using ITS1/ITS4 primer pair [16]. The amplified products of dermatophytes and yeasts were then respectively digested with the restriction enzymes *Mva*I and *Msp*I in a 30-μL reaction mixture according to the *manufacturer's instructions* *(*Thermo Fisher Scientific, Waltham, MA, USA). The restriction products were separated by electrophoresis on 2% agarose gel, stained with ethidium bromide, visualized under UV light and photographed. 

For the identification of isolates, the banding profile of each colony was compared with the specific banding patterns demonstrated in the previous reports for dermatophyte [17] and yeast species [18]. For the identification of NDMs and yeasts other than *Candida*, depending on the results of morphological assessments, ITS1-5.8S-ITS2 regions of rDNA and beta-tubulin locus were subjected to sequencing by ITS1/ITS4 and Bt2a/Bt2b primer pairs [19], respectively. The obtained sequences were edited and compared to other publicly available sequences through the Westerdijk Fungal Biodiversity Institute database (formerly known as the Fungal Biodiversity Center/CBS).

***Statistical analysis***

All the descriptive data were collected in Microsoft Excel and imported to SPSS version 20.0. The correlations of gender and causative agent with site of involvement (toenail or fingernail) and age with site of infection were respectively examined by Chi-square (x^2^) and Mann-Whitney tests. *P-value* less than 0.05 was considered statistically significant.

## Results

Of the 433 patients enrolled in the study, onychomycosis was confirmed in 154 cases (35.6%). Twenty-one of these cases were diagnosed only by direct microscopic examination, 132 cases by both direct examination and culture, and 1 case only by culture. The patients consisted of 56 males (36.4%) and 98 females (63.6%), whose age ranged from 2-85 years (mean age: 44 years), and the prevalence of infection was the highest in the 41-50 years age group. Tables 1 and 2 present the frequency of onychomycosis in relation to gender, site of involvement, causative agent, and age. Infection more commonly occurred due to yeasts (57.15%); with *C. albicans* as the most common species (29.35%), followed by *C. parapsilosis* (13.8%), *C. tropicalis* (4.5%), *C. krusei* (3%), *C. glabrata* (2.25%), and other yeast species (2.25%; Table 3). Dermatophytes were isolated from 38.35% of the cases; the most commonly isolated dermatophytes were found to be *T. interdigitale* (21.1%), *E. floccosum* (10.5%), *T. rubrum* (5.25%), and *M. canis* (1.5%). Non-dermatophytic molds were the least frequent agents, which were isolated only from 4.5% of positive cultures and included *Aspergillus* spp., (2.25%), *Chaetomium subglobosum*, *Aphanoascus verrucosus*, and *Penicillium ruber* (each one 0.75%). The type of infectious agent had a significant effect on thesite of involvement (*P<0.05*), indicating that onychomycosis caused by dermatophytes (73.2%) and NDMs (83.3%) was more frequently seen in toenails, whereas yeasts caused infection mostly in fingernails (78.3%). Gender had a significant impact on the site of infection (*P<0.05*), indicating that fingernail onychomycosis was significantly more prevalent in females than in males (67 vs. 31 cases), while toenail infection was significantly more common in males than in females (35 vs. 21 cases). The correlation between age and prevalence of onychomycosis was statistically significant, and toenail infection was more prevalent in higher age groups (*P<0.05*).

**Table 1:** Distribution of onychomycosis according to the site of infection and age group

**Age groups**	**Site of involvement**		
	**Toenail**	**Fingernail**	**Total (%)**
<10	0	5	5 (3.2)
11-20	1	7	8 (5.3)
21-30	8	13	21 (13.6)
31-40	10	21	31 (20.1)
41-50	14	26	40 (26)
> 50	33	16	49 (31.8)
Total (%)	66 (42.9)	88 (57.1)	154 (100)

**Table 2:** Distribution of onychomycosis in relation to gender, etiologic agent, and site of involvement

	**Site of involvement**		
	**Toenail (%)**	**Fingernail (%)**	**Total (%)**
**Sex**			
Male	35 (62.5)	21 (37.5)	56 (36.4)
Female	31 (31.6)	67 (68.4)	98 (63.6)
Total	66 (42.9%)	88 (57.1)	154 (100)
**Causative agent**			
Dermatophytes	41 (73.2)	15 (26.8)	56 (36.4)
Yeasts	20 (21.7)	72 (78.3)	92 (59.7)
Non-dermatophytic molds	5 (83.3)	1 (16.7)	6 (3.9)
Total	66	88	154 (100)

**Table 3:** Results of onychomycosis agents identification in 133 culture-positive cases

**Causative agent**		**Identification method**	**Frequency**	**%**
Dermatophytes	*T. interdigitale*	ITS-RFLP	28	21.1
	*E. floccosum*	ITS-RFLP	14	10.5
	*M. canis*	ITS-RFLP	2	1.5
	*T. rubrum*	ITS-RFLP	7	5.25
	Total		51	38.35
Yeasts	*C. albicans*	ITS-RFLP	39	29.35
	*C. tropicalis*	ITS-RFLP	6	4.5
	*C. glabrata*	ITS-RFLP	3	2.25
	*C. parapsilosis*	ITS-RFLP	17	12.8
	*C. krusei*	ITS-RFLP	4	3.0
	*C. guilliermondii*	ITS-RFLP	1	0.75
	*Aureobasidium pullulans*	ITS-sequencing	1	0.75
	*Trichosporon asahii*	ITS-sequencing	1	0.75
	Mixed yeast infection	ITS-RFLP	4	3.0
	Total		76	57.15
Non-dermatophytic molds	*Chaetomium subglobosum*	ITS-sequencing	1	0.75
	*Aphanoascus verrucosus*	ITS-sequencing	1	0.75
	*Penicillum ruber*	BT2-sequencing	1	0.75
	*Aspergillus terreus*	BT2-sequencing	1	0.75
	*Aspergillus niger*	BT2-sequencing	1	0.75
	*Aspergillus flavus*	BT2-sequencing	1	0.75
	Total	-	6	4.5
	Total	-	133	100.0

## Discussion

Onychomycosis is a prevalent infection in Iran [9-14], but there is a scarcity of comprehensive assessments of onychomycosis in Khuzestan Province, southwest of Iran. To the best of our knowledge, this study provided the first dataset regarding the clinical and mycological aspects of this infection in this area of Iran. The prevalence rate of onychomycosis in this study (35.6%) was almost similar to that described from Isfahan Province, Iran [10], but it was less than the prevalence rates of 40.2-45% reported in other parts of the country [9, 12, 13], 68.8% from Taiwan [20], 42% from Brazil [21], 50.8% from Serbia [22], and 82% from Saudi Arabia [23].

 Onychomycosis is known to occur at any age, but more frequently in the 40+ years age group and less commonly prior to puberty [4]. In line with this fact and results of several former studies [9, 21, 23, 24], the majority of the patients (57.8%) in this study were in the age groups of 41-50 and >50 years, which was statistically significant. A large number of patients in this study (33.7%) were aged between 21 to 40 years. This could be due to the fact that fungal nail infections might be assumed as a worthless cosmetic dilemma rather than a disease in this region; therefore, since younger patients are more conscious of their appearance, they visit clinics for treatment. There were only five children (< 10 years) with nail infection, accentuating the fact that this infection is unusual in this age group, even in our community. Contrary to some reports from the Middle East, southwest Asia, and India [5, 20, 25] and in agreement with some previous reports from Europe [22, 24], South America [21], and Iran [9, 11, 12-14], females were predominantly more affected by onychomycosis (63.6%) than males in Khuzestan Province. An explanation for this finding is that for any nail disorder, females are normally more likely to present to clinics for clinical investigation and treatment. Additionally, this finding of the current survey was consistent with the results of other studies denoting that the higher involvement of fingernail onychomycosis in females (78.3% in this study) is associated with *Candida *species as the main causative agents [5, 10, 12-14, 25]. On the other hand, the data inferred from the current survey strongly support that onychomycosis of toenails mainly occurs in men and is caused by dermatophytes and NDMs [10, 11, 13, 14, 20, 25].

In view of etiology, yeasts were the main infectious agents in Khuzestan Province and in fingernail onychomycosis, of which *C. albicans*, *C. parapsilosis*, and *C. tropicalis* were the most common species, respectively. Such results were in agreement with reports from Brazil, India, Saudi Arabia, and Iran [5, 9, 10, 21, 23]. The frequency of *Candida albicans* (50%) as the main *Candida* species causing onychomycosis in this study was lower than 58-68% reported from Qazvin and Isfahan provinces [9, 11], but was higher than 23-45% from Shiraz, Tehran, and Kermanshah [12, 13, 26]. 

As shown in the present study, the new scenario for *Candida* onychomycosis in Iran is the growing incidence of onychomycosis by non-*albicans Candida* species (NACS) such as *C. parapsilosis*, *C. tropicalis, C. glabrata*, and *C. krusei* [9, 26]. Review of the literature revealed that most of the previous descriptive studies were performed by conventional unreliable procedures for the identification of onychomycosis agents, and the identity of most NACS isolates was narrated as *Candida* spp. [12-14]; therefore, it is difficult to estimate the true incidence of NACS in *Candida* onychomycosis. However, in the present study, all the yeast isolates were characterized to the species level by sequence-based methods. *Candida parapsilosis* comprised 22% of all yeast isolates in our study, and in two recent molecular-based screenings from Isfahan and Shiraz, the isolation rates of *Candida* species from nail infections were reported as 13% and 44%, respectively [11, 26]. Generally, infection by NACS is of particular importance due to the intrinsic or acquired resistance of these agents to antifungal drugs of the azole and echinocandin classes [27, 28], underlining the importance of the accurate identification of *Candida* clinical isolates to the species level to have a successful treatment. The two strains of *Aureobasidium pullulans *and *Trichosporon asahii, *which are reported for the first time from onychomycosis in Iran, were identified by sequencing.

Dermatophytes comprised the second most prevalent group of onychomycosis agents among Khuzestanian patients, which mostly infected toenails (73.2%). While *Trichophyton rubrum* was by far the most common species causing nail infection [1, 21], recent studies indicated that *T. interdigitale* (*T. mentagrophytes*) is the predominant causative agent in Iran [11, 14, 15, 29]. This finding was also reflected in the current survey, where *T. interdigitale* (54.9%), followed by *E. floccosum* (27%) and *T. rubrum* (13%), encompassed the dominant species causing onychomycosis, respectively. These findings were in line with those reported from Isfahan, Ahvaz, and Tehran [11, 15, 29], but distinct from results of other studies where *T. rubrum* was the second prevalent species depending on the time of study, geographic location, and method of identification [9, 13]. 

Review of the literature and the data from this study point out that contrary to the popular belief emphasizing on the dermatophytes as the leading causes of onychomycosis worldwide, it is at least from one decade ago that yeasts (alone or in combination with NDMs) are the main agents of onychomycosis in most areas of Iran [11, 12-14, 30], and even other parts of the world [5, 21, 23].

The frequencies of NDMs as the agents of onychomycosis in some reports from Iran varied from 2.9% to 22% [10-14]. In this investigation, NDMs constituted the least significant portion of agents causing infection (3.9%), among which *Aspergillus* spp. were the most frequent agents. Nevertheless, another series of studies from Iran and other countries have recently reported the overtaking of NDMs as the emerging causes of onychomycosis [6, 30, 31]. Currently, it seems that members of the *Aspergillus* genus are the main NDMs causing onychomycosis in Iran [6, 10, 13, 14, 30]. 

Given that like *Candida* spp., the intrinsic or acquired resistance to azoles is observed in *Aspergillus* species, the differentiation of these filamentous agents from dermatophytes is essential for providing precise treatments and improving affected patients [29]. It is worth mentioning that filamentous fungi, namely *Chaetomium subglobosum*, *Aphanoascus verrucosus*, and *Penicillium ruber*, have been isolated for the first time from infected nails in this study, and thus, the role of these NDMs as less frequent agents of onychomycosis should not be ignored.

It has been mentioned that the etiology of onychomycosis reported in different studies could be influenced most probably by clinical types of onychomycosis and the population (by sex) included in each study, but not by climate or geographic conditions [10]. In the other words, in studies wherein a large proportion of fingernails or housewives (females) were included, yeasts were found to be the more frequent agents, and in contrast, those that included toenails showed dermatophytes and NDMs as the dominant agents. However, in our study, both suspected men and women were included, and the ratio of sampled individuals (33% males and 67% females) to confirmed onychomycosis cases (36.4% males vs. 63.4% females) was not statistically significant, indicating a shift from dermatophytes towards yeasts as the leading agents of onychomycosis. Overall, the reason for the gender discrepancy in the frequency of onychomycosis is inconspicuous, and further large-scale studies are required to investigate this issue.

## Conclusion

Our findings greatly support the fact that onychomycosis remains as a never-ending problem and that dermatophytes and NDMs play a major role in toenail onychomycosis, whereas yeasts tend to cause onychomycosis in fingernails. Meanwhile, this study highlighted that the causative agents of onychomycosis have shifted from dermatophytes to yeasts in Khuzestan Province.
